# Human CD22-Transgenic, Primary Murine Lymphoma Challenges Immunotherapies in Organ-Specific Tumor Microenvironments

**DOI:** 10.3390/ijms221910433

**Published:** 2021-09-28

**Authors:** Franziska Gsottberger, Carolin Brandl, Kerstin Wendland, Srdjan Petkovic, Charlotte Emmerich, Ramona Erber, Carol Geppert, Arndt Hartmann, Andreas Mackensen, Lars Nitschke, Fabian Müller

**Affiliations:** 1 Department of Internal Medicine 5, Haematology and Oncology, University Hospital of Erlangen, Friedrich-Alexander University of Erlangen-Nuremberg (FAU), 91054 Erlangen, Germany; franziska.gsottberger@uk-erlangen.de (F.G.); kerstin.wendland@uk-erlangen.de (K.W.); srdjan.petkovic@uk-erlangen.de (S.P.); charlottewagner19@gmail.com (C.E.); Andreas.Mackensen@uk-erlangen.de (A.M.); 2 Department of Biology, Friedrich-Alexander University of Erlangen-Nuremberg (FAU), 91054 Erlangen, Germany; carolin.brandl@uk-erlangen.de (C.B.); lars.nitschke@fau.de (L.N.); 3 Department of Pathology, University Hospital of Erlangen, Friedrich-Alexander University of Erlangen-Nuremberg (FAU), 91054 Erlangen, Germany; ramona.erber@uk-erlangen.de (R.E.); carol.geppert@uk-erlangen.de (C.G.); arndt.hartmann@uk-erlangen.de (A.H.)

**Keywords:** *myc*-driven lymphoma, mouse model, lymphoma microenvironment, tumor microenvironment, CD22, immunotoxin, checkpoint molecule, PD-L1, myeloid derived suppressor cells, MDSCs

## Abstract

Targeted immunotherapies have greatly changed treatment of patients with B cell malignancies. To further enhance immunotherapies, research increasingly focuses on the tumor microenvironment (TME), which differs considerably by organ site. However, immunocompetent mouse models of disease to study immunotherapies targeting human molecules within organ-specific TME are surprisingly rare. We developed a *myc*-driven, primary murine lymphoma model expressing a human-mouse chimeric CD22 (h/mCD22). Stable engraftment of three distinct h/mCD22^+^ lymphoma was established after subcutaneous and systemic injection. However, only systemic lymphoma showed immune infiltration that reflected human disease. In this model, myeloid cells supported lymphoma growth and showed a phenotype of myeloid-derived suppressor cells. The human CD22-targeted immunotoxin Moxetumomab was highly active against h/mCD22^+^ lymphoma and similarly reduced infiltration of bone marrow and spleen of all three models up to 90-fold while efficacy against lymphoma in lymph nodes varied substantially, highlighting relevance of organ-specific TME. As in human aggressive lymphoma, anti-PD-L1 as monotherapy was not efficient. However, anti-PD-L1 enhanced efficacy of Moxetumomab suggesting potential for future clinical application. The novel model system of h/mCD22^+^ lymphoma provides a unique platform to test targeted immunotherapies and may be amenable for other human B cell targets such as CD19 and CD20.

## 1. Introduction

While cancer research has focused for many years primarily on tumor cells, emerging novel concepts increasingly address the crosstalk between tumor cells and the tumor microenvironment (TME). The TME consists of immune cells, stromal cells, blood vessels, and extracellular matrix that interact in complex networks by direct cell-to-cell contact and by soluble factors including metabolites and cytokines [1,2]. Composition of the TME strongly varies between tumors of distinct individuals, but also within one patient with multiple tumor sites [3,4]. In virtually all malignancies, subsets of myeloid cells in the TME such as monocytes, macrophages, neutrophils, or myeloid-derived suppressor cells (MDSC) have been recognized as promoters of tumor growth, immune evasion, and drug resistance [5]. In line, relevance of myeloid cells, predominantly MDSCs, have been well described for indolent and aggressive lymphoma including diffuse large B cell lymphoma (DLBCL) or B cell acute lymphoblastic leukemia (B ALL) [6,7,8,9,10]. MDSCs are a heterogeneous population of immature myeloid cells that potently inhibit T cell function and induce an exhausted T cell phenotype including an upregulation of PD-1/PD-L1 in mice and men [11,12]. A high rate of MDSCs at first diagnosis correlates with poor response to standard treatment, shorter progression-free survival, and shorter overall survival for patients with DLBCL as well as for other lymphoma [13,14,15,16]. The role of TME in relapse or in treatment resistance and ways to overcome possible TME-induced resistance are deduced from observational studies in men and from in vivo model systems with functional immune systems [17].

With the goal of further improving treatment outcome, various targeted therapies against cancer antigens have been developed. An attractive target antigen on B cell malignancies is the surface antigen CD22 [18]. CD22 is highly expressed on various B cell malignancies including B ALL and DLBCL [18]. Already approved drugs and drugs in clinical development against CD22 include chimeric antigen receptor (CAR) T cells [19,20], bispecific T cell engagers (BiTEs) [21], antibody drug conjugates (ADC, e.g., Inotuzumab Ozogamicin) [22], and recombinant immunotoxins (e.g., Moxetumomab pasudotox, Lumoxiti^®^, Moxe) [23]. Moxetumomab consists of a CD22 targeting dsFv fused to a 38 kDa fragment of *Pseudomonas* exotoxin A [23]. After surface binding and internalization, Moxetumomab traverses various intracellular compartments before it APD-ribosylates eukaryotic elongation factor 2 (eEF2). This enzymatic reaction arrests protein synthesis and induces cell death [23]. All current CD22-targeted drugs detect human but not murine CD22 and thus have been studied exclusively in immunocompromised xenograft mouse models. Aiming to establish human CD22 in mice, a fully human CD22 did not completely support murine CD22 function and B cell development [24]. Hence, we utilized a chimeric version composed of the human extracellular and the murine intracellular domains of CD22 (h/mCD22). This CD22-chimera supports normal murine B cell function and leads to a near physiologic h/mCD22 expression among murine B cell subsets [25]. Aiming for mice that develop primary h/mCD22^+^ lymphoma, we chose from various possible oncogenes the B cell specific overexpression of c-MYC that leads to spontaneous aggressive lymphoma [26,27,28,29]. Here, we describe models of three distinct, serially transplanted *myc*-driven and h/mCD22^+^ lymphoma in immunocompetent TME of C57BL/6 (BL6) mice in which organ-specific TME and efficacy of targeted immunotherapies was characterized.

## 2. Results

### 2.1. Development of a Myc-Driven, h/mCD22 Expressing, Primary Lymphoma Model

**Figure 1:**
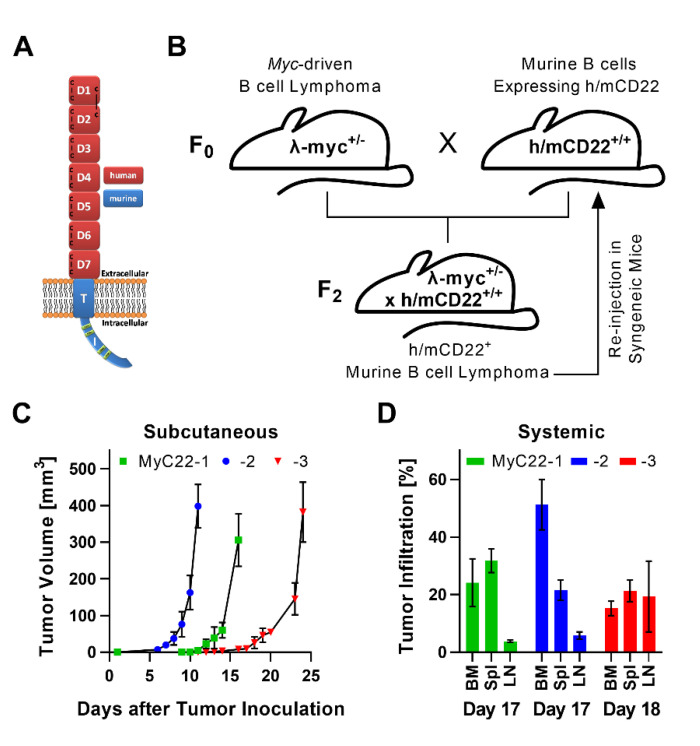
Stable engraftment of *myc*-driven h/mCD22 expressing, primary lymphoma in syngeneic mice. (**A**) h/mCD22 consists of human (red) extracellular and murine (blue) intracellular domains of CD22. (**B**) Breeding scheme to generate mice spontaneously developing h/mCD22^+^ B cell lymphoma that were reinjected in syngeneic mice. (**C**,**D**) Engraftment of three distinct primary lymphoma termed MyC22-1, -2, -3. (**C**) Ten million lymphoma cells were subcutaneously injected on day one. Tumor volume was measured as length × width^2^ × 0.4. (**D**) One million lymphoma cells were intravenously injected on day one. Bone marrow (BM), spleen (Spl), and lymph nodes (LN) were extracted at the indicated days and tumor infiltration was analyzed by flow cytometry after staining with anti-human CD22 and anti-mouse CD19. Shown is the mean of n = 3 mice at each time point. Error as SD.

To establish a human CD22^+^ lymphoma mouse model, mice carrying the chimeric h/mCD22 (Figure 1A) were crossbred with λ-myc mice. Mice homozygous for h/mCD22 and heterozygous for λ-myc developed systemic lymphadenopathy, splenomegaly, and bone marrow infiltration of a monomorphic h/mCD22^+^ B cell population between five to eight weeks of age (Figure S1). The monomorphic population was isolated from three individual mice and the distinct *myc*-driven and h/mCD22^+^ clones were named chronologically MyC22-1, -2, -3. A single cell suspension of each clone was transplanted into individual sets of h/mCD22 mice (Figure 1B). Mice were injected either subcutaneously (s.c.) or intravenously (i.v., from here called systemically). All mice showed successful engraftment of the same B cell population as seen in parental mice within days, thus fulfilling all criteria of an aggressive B cell lymphoma (Figure 1C,D and Figure S1). All three clones stably engrafted in all mice injected and were not rejected. Growth kinetics and rate of lymphoma infiltration by organ differed among the clones (Figure 1C,D). The time until s.c. tumors reached a size of 300–400 mm^3^ was on average 16 days for MyC22-1, 10 days for MyC22-2, and 24 days for MyC22-3 (Figure 1C). Distinct growth kinetics for each lymphoma clone were also found in the systemic model (Figure 1D). On day 17, MyC22-1 showed tumor infiltration of 24.1% in bone marrow (BM), 31.7% in spleen, and 3.8% in lymph nodes (LN). On the same day, MyC22-2 infiltrated BM by 51.2%, spleen by 21.6%, and LN by 5.8%. MyC22-3 showed an equally distributed tumor infiltration of 15.2% in BM, 21.3% in spleen, and 19.3% in LN on day 18 (Figure 1D). Differences in growth suggest that every clone represents a unique lymphoma model despite sharing c-MYC overexpression as main driver.

**Figure 2:**
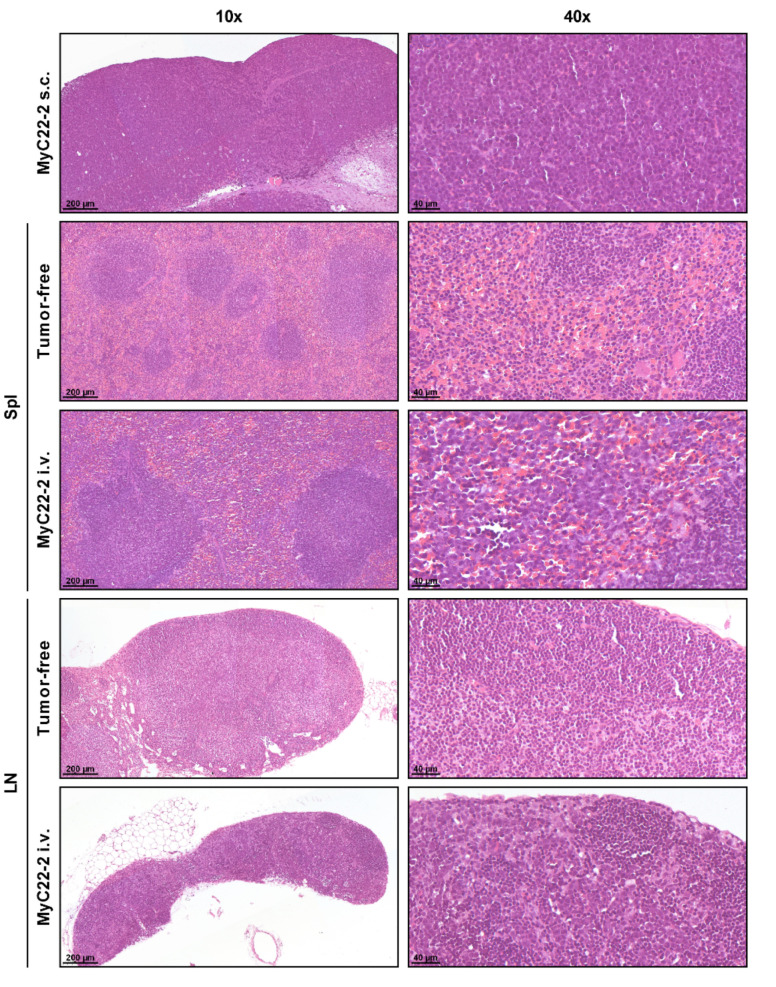
Histological characterization of lymphoma-infiltrated organs. Subcutaneous MyC22-2 tumors were extracted at a size of ~400 mm^3^. Systemically infiltrated spleen (Spl) and lymph nodes (LN) were extracted on day 15 or from age-matched tumor-free mice. Shown are representative parts of indicated organs stained with hematoxylin and eosin (H&E) at a 10-fold and a 40-fold magnification.

Flow cytometry and histology confirmed that s.c. tumors of MyC22-1, -2, and -3 at a size of 300–400 mm^3^ consisted of more than 95% of tumor cells (Figure 2, Figures S2 and S3). In contrast, histopathologic examination of systemic lymphoma showed diffuse infiltration of spleen and LN (Figure 2 and Figure S3). On day 15 after systemic injection of MyC22-2, the spleen showed diffuse infiltration of large lymphocytic cells while physiologic architecture including lymphoid follicles, as found in tumor-free tissue, was maintained. Similarly, LNs were diffusely infiltrated presenting with a less pronounced differentiation between cortex and medulla compared to LNs of healthy mice. Remaining physiologic organ architecture suggested infiltration of organ-specific immune cells within systemic tumors, whereas s.c. tumors were dominated by tumor cells.

**Figure 3:**
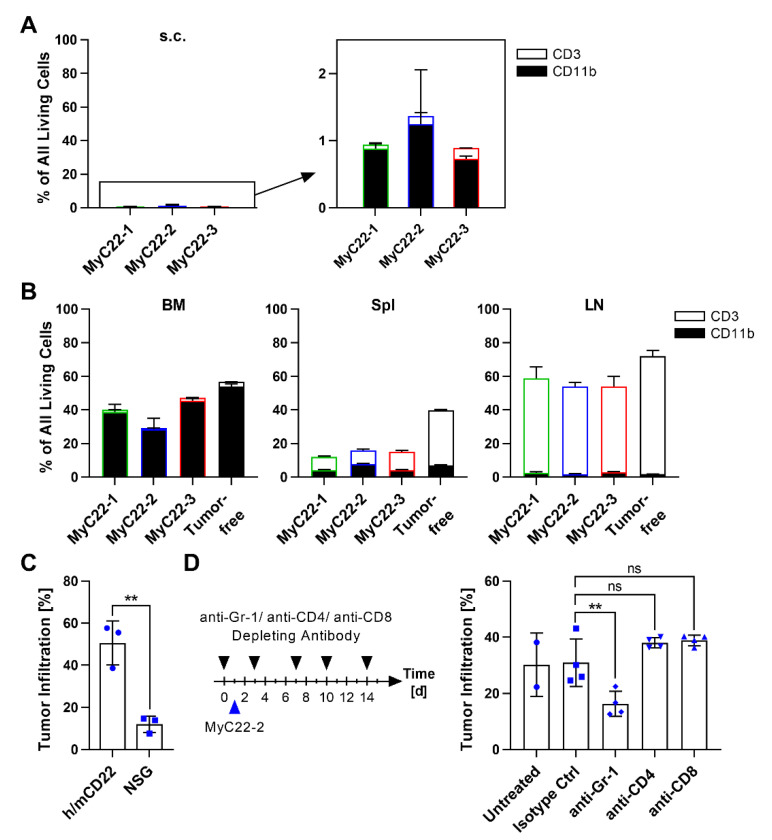
Characterization of immune cells of lymphoma infiltrated organs and their relevance for lymphoma growth. (**A**,**B**) Subcutaneous tumors (s.c.) (**A**) and bone marrow (BM), spleen (Spl), and lymph nodes (LN) of systemically injected MyC22 (**B**) were analyzed by flow cytometry for infiltration of CD3^+^ and CD11b^+^ cells compared to tumor-free mice. Bars show means of n = 3 mice. Error as SD. (**C**) One million MyC22-2 cells were intravenously injected in h/mCD22 and NSG mice. On day 15, bone marrow was analyzed by flow cytometry after staining with anti-human CD22 and anti-mouse CD19. Shown is the percentage of tumor infiltration of all living cells in BM. Bars show means of n = 3 mice, each symbol represents an individual mouse. Shown are representative results of two independent experiments. Error as SD. *p*-values were determined by unpaired *t*-test. **: *p* ≤ 0.01. (**D**) Mice were i.p. injected with 200 µg anti-Gr-1, anti-CD4, and anti-CD8 antibodies or isotype control on days 0, 3, 7, 10, 14. On day one, one million MyC22-2 cells were injected intravenously. Tumor infiltration of bone marrow was analyzed on day 15 as described in (C). Shown is the percentage of tumor infiltration of all living cells in BM. Bars show means of n = 2–4 mice, each symbol represents an individual mouse. Error as SD. *p*-values were determined by one-way ANOVA (Dunnett’s test). Not significant (ns): *p* > 0.05, **: *p* ≤ 0.01.

We further analyzed organ-specific immune cell infiltration by phenotype using flow cytometry (Figure 3A,B). All three s.c. tumors contained few immune cells. MyC22-1 s.c. tumors were infiltrated by 0.9% CD11b^+^ myeloid cells and 0.06% CD3^+^ T cells. MyC22-2 s.c. tumors showed 1.2% CD11b^+^ and 0.1% T cell infiltration, and MyC22-3 displayed 0.7% CD11b^+^ and 0.2% T cell infiltration (Figure 3A). In contrast to the scarce infiltration of s.c. tumors, the composition of immune cells of systemic lymphoma resembled the immune composition of the respective healthy organ (Figure 3B). In BM, myeloid cells predominated with 38.8% in MyC22-1, 28.4% in MyC22-2, and 45.6% in MyC22-3, while T cells represented only 0.8% to 1.7% of all immune cells in the three models. The spleen was infiltrated by 4.3% to 7.7% myeloid cells and by 7.7% to 10.9% CD3^+^ T cells. LNs of all three models were infiltrated by 1.8% to 2.9% myeloid cells and by more than 50% of CD3^+^ T cells. Since B cell lymphoma in men commonly show substantial immune cell infiltration and mostly grow in lymphoid organs, but only rarely in the skin, the systemic model recapitulated the human disease more closely and was therefore characterized in more detail.

### 2.2. Myeloid Cells Support Tumor Growth and Contribute to an Immunosuppressive TME

Next, we tested the effect of immune cells on tumor growth kinetics using the fastest growing clone MyC22-2. We compared BM lymphoma infiltration rate of immunocompetent h/mCD22 mice (BL6 background) and of immunocompromised NSG mice (lacking functional T cells, NK cells, and myeloid cells). On day 15, BM infiltration of NSG mice was 12.1%, which was significantly less than 50.6% BM infiltration of h/mCD22 mice (*p* = 0.0039, Figure 3C). The higher infiltration rate in immunocompetent mice suggests that immune cells may enhance BM lymphoma growth. To dissect the impact of distinct immune cell subsets, we individually depleted CD4^+^ T cells, CD8^+^ T cells, and Gr-1^+^ myeloid cells in MyC22-2-bearing mice, respectively (Figure 3D). On day 15, BM infiltration rate was similar after depletion of CD4^+^ or CD8^+^ T cells, whereas depletion of Gr-1^+^ myeloid cells significantly reduced tumor infiltration by 1.9-fold from 31.0% to 16.3% compared to isotype control (*p* = 0.0034, Figure 3D). These data suggest that myeloid cells enhance growth of MyC22-2 lymphoma in vivo.

**Figure 4:**
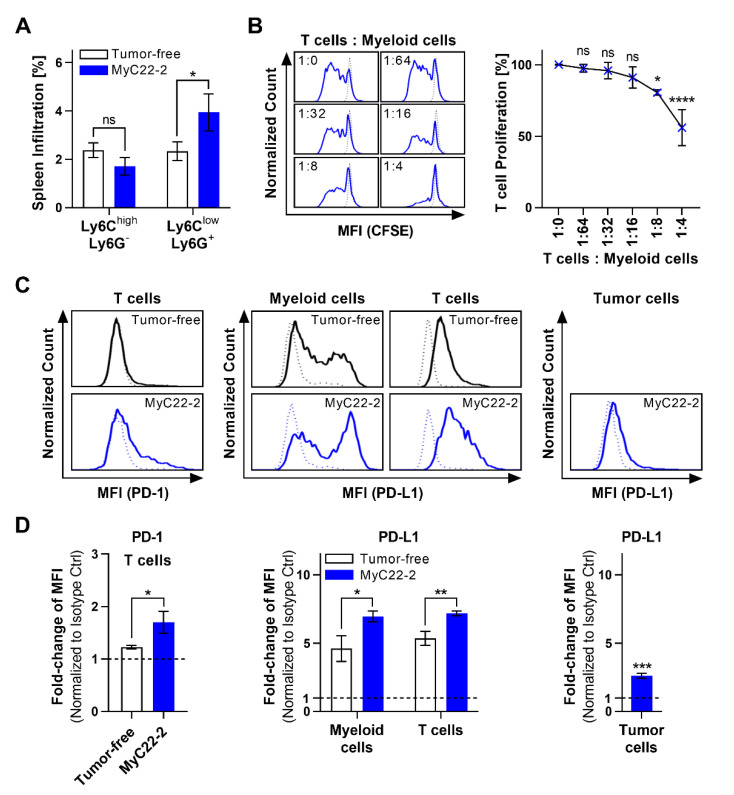
Inhibitory myeloid cells contribute to an immunosuppressive environment. (**A**) CD11b^+^ myeloid cells from spleen of MyC22-2-bearing mice on day 15 and of healthy mice were characterized by flow cytometry after staining for Ly6C and Ly6G. Bars show means of n = 3 mice. Shown are representative results of at least two independent experiments. Error as SD. *p*-values were determined by unpaired *t*-test. Not significant (ns): *p* > 0.05, *: *p* ≤ 0.05. (**B**) Healthy CD4^+^ T cells were co-cultured with myeloid cells isolated from spleens of mice bearing MyC22-2 at the indicated ratios. T cells were CFSE-stained and stimulated with anti-CD3/-CD28 beads and proliferation was analyzed by flow cytometry after 72 h of co-culture. Shown are representative CFSE histograms of CD4^+^ T cells at indicated conditions (blue) compared to unstimulated T cells (dashed line). T cell proliferation after myeloid co-culture according to CFSE signal was normalized to T cells only. Shown is the mean of n = 3 biological replicates. Error as SD. *p*-values were determined by one-way ANOVA (Dunnett’s test). Not significant (ns): *p* > 0.05, *: *p* ≤ 0.05, ****: *p* ≤ 0.0001. (**C**,**D**) Spleens from MyC22-2-bearing (blue) and tumor-free mice (black) were stained for PD-1 and/or PD-L1 on CD3^+^ T cells, CD11b^+^ myeloid cells, and CD19^+^ tumor cells and analyzed by flow cytometry. (**C**) Shows exemplary histograms for PD-1 and PD-L1 signal (solid line) compared to isotype control (dotted line). (**D**) Shows fold-change of PD-1 and PD-L1 mean fluorescence intensity (MFI) compared to isotype control. Bars represent the mean of n = 3 mice, error as SD. *p*-values were determined by unpaired *t*-test. *: *p* ≤ 0.05, **: *p* ≤ 0.01, ***: *p* ≤ 0.001.

We further characterized myeloid cells in the MyC22-2 model. Phenotypical analysis showed that CD11b^+^ cells were predominated by monocytic Ly6C^high^ Ly6G^−^ and granulocytic Ly6C^low^ Ly6G^+^ cells. The granulocytic subset was enriched in MyC22-2 tumor-bearing mice compared to healthy mice (Figure 4A). To prove their immune inhibitory function and thus fulfill definition of MDSCs [30], CD11b^+^ myeloid cells isolated from lymphoma-bearing spleen were co-cultured with healthy T cells. Increasing numbers of myeloid cells blocked cell division of T cells by up to 45% in a cell dose-dependent manner (Figure 4B). To probe for functional immune inhibition in vivo, we additionally analyzed PD-1 and PD-L1 surface expression levels of various cell types within the lymphoma TME. PD-1 expression of T cells from spleen of MyC22-2-bearing mice was 1.4-fold higher compared to tumor-free mice (Figure 4C,D). In healthy mice, thus at baseline, PD-L1 levels of spleen-derived myeloid cells were 4.6-fold and of T cells 5.4-fold over background as defined by isotype control (Figure 4C,D). Compared with healthy controls, PD-L1 expression of spleen-derived myeloid cells and T cells in tumor-bearing mice increased by 1.5-fold (*p* = 0.0163) and by 1.3-fold (*p* = 0.0042), respectively. Lymphoma cells themselves displayed an increase of PD-L1 surface expression by 2.6-fold compared to isotype control (*p* = 0.0001, Figure 4C,D). The significant increase of PD-1/PD-L1 checkpoint molecules on various cell subsets together with the presence of MDSCs indicated an immunosuppressive TME of mice bearing MyC22-2.

### 2.3. PD-L1 Checkpoint Blockade Shows Limited Efficacy against MyC22

**Figure 5:**
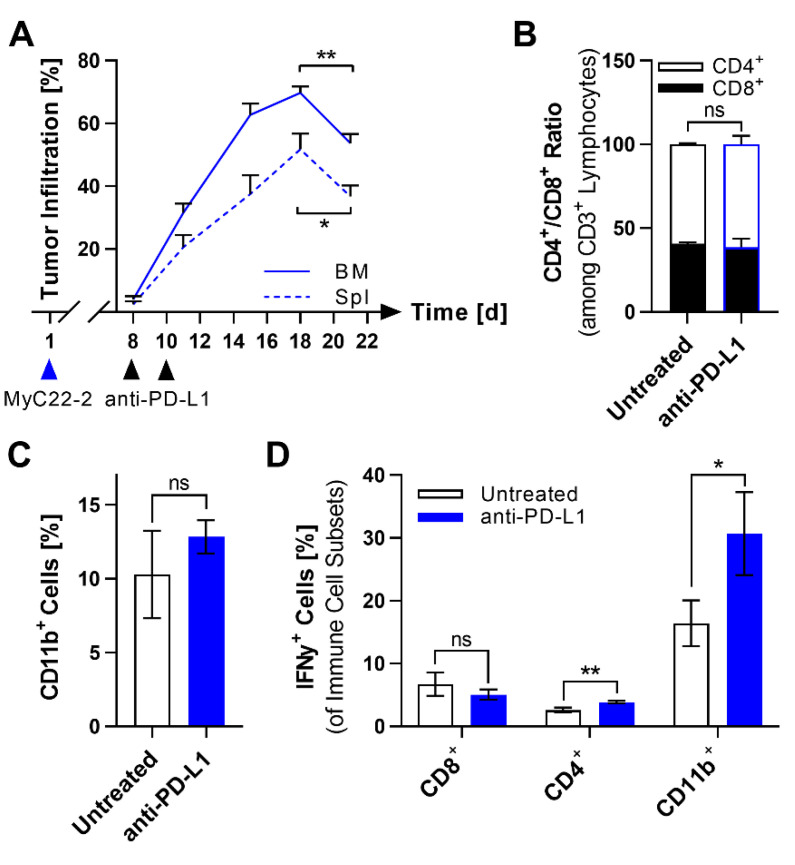
Reduction of tumor infiltration after anti-PD-L1 monotherapy is accompanied by myeloid cell activation. (**A**) One million cells of MyC22-2 were intravenously injected on day one. At the indicated days, 200 µg anti-PD-L1 was given intraperitoneally. Tumor infiltration was monitored in bone marrow (BM) and spleen (Spl) by flow cytometry after staining with anti-human CD22 and anti-mouse CD19. Bars show means of n = 3 mice at each time point. Error as SD. *p*-values were determined by unpaired *t*-test. *: *p* ≤ 0.05, **: *p* ≤ 0.01. (**B**–**D**) Spleens of MyC22-2-bearing mice were analyzed by flow cytometry 10 days after anti-PD-L1 treatment and compared to untreated lymphoma-bearing mice. Shown is the ratio of CD4^+^/CD8^+^ T cells (**B**), percentage of CD11b^+^ myeloid cells (**C**), and IFNγ expression among indicated immune cell subsets (**D**). Each bar shows the mean of n = 3 mice. Error as SD. *p*-values were determined by unpaired *t*-test. Not significant (ns): *p* > 0.05, *: *p* ≤ 0.05, **: *p* ≤ 0.01.

Based on the PD-L1 upregulation, we hypothesized that therapy with anti-PD-L1 antibodies may have good efficacy. Mice bearing systemic MyC22-2 tumors were treated with two intraperitoneal (i.p.) doses of anti-PD-L1. At various time points after treatment, mice were sacrificed and analyzed for lymphoma infiltration. Up to day 18, treated mice showed an increase in tumor burden (Figure 5A). However, on day 21, 13 days after the first dose of anti-PD-L1, MyC22-2 infiltration significantly decreased in spleen by 15.0% (*p* = 0.0125) and in BM by 16.0% (*p* = 0.0016, Figure 5A). Because the time-delayed response of 13 days post anti-PD-L1 indicated immune activation, we analyzed spleens of mice bearing MyC22-2 10 days after the first dose of anti-PD-L1 for immunological changes. However, neither the CD4/CD8 T cell ratio nor the myeloid cell infiltration rate changed after anti-PD-L1 treatment (Figure 5B,C). To test cell functionality, we assessed intracellular IFNγ expression by T cell and myeloid cell subsets after anti-PD-L1 therapy compared to untreated control. The frequency of IFNγ^+^ myeloid cells doubled to 30.7% (*p* = 0.0308), the rate of IFNγ^+^ CD4^+^ T cells increased by 1.3-fold (*p* = 0.0069), and the rate of IFNγ^+^ CD8^+^ T cells did not change compared with control mice (Figure 5D). As in men [31], anti-PD-L1 therapy alone only showed little efficacy against MyC22-2 and did not activate CD8^+^ T cells.

### 2.4. Efficacy of Moxetumomab Was Clone and Organ Dependent

**Figure 6:**
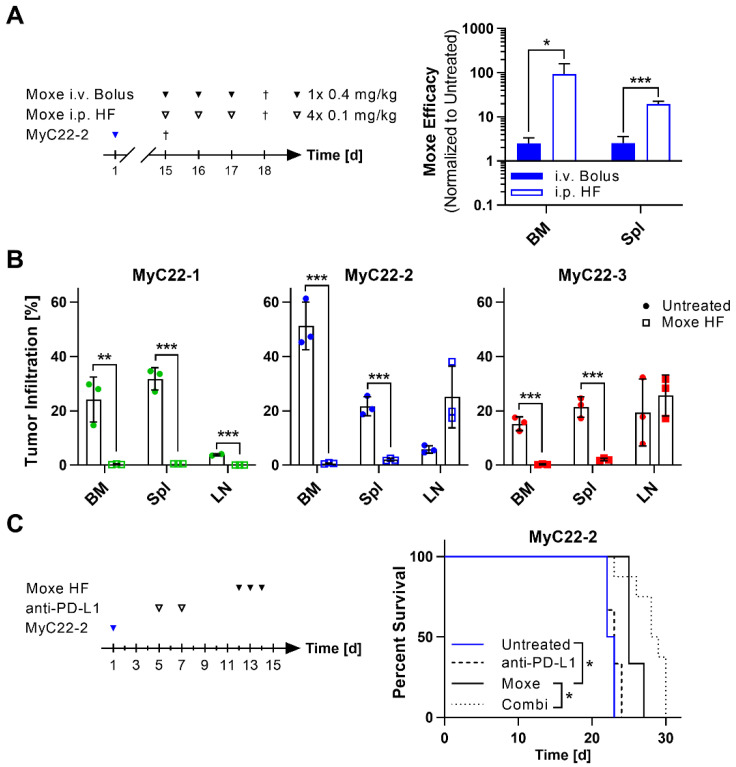
CD22-targeted therapy with Moxetumomab shows organ-dependent activity against h/mCD22 positive lymphoma and is enhanced by immune checkpoint blockade. (**A**) One million cells of MyC22-2 were intravenously injected on day one. At the indicated time points, mice received either one bolus dose of 0.4 mg/kg Moxetumomab (Moxe) intravenously (i.v.) or were treated at high frequency (HF) with four doses of 0.1 mg/kg Moxetumomab intraperitoneally (i.p.). Untreated mice were euthanized at treatment start and treated mice one day after last injection (†). Bone marrow (BM) and spleen (Spl) of Moxetumomab treated and untreated mice were analyzed by flow cytometry after staining with anti-human CD22 and anti-mouse CD19. Moxetumomab efficacy is defined as the inverse fold-change of tumor infiltration normalized to untreated. Bars show means of n = 3 mice. Shown are representative results of at least two independent experiments. Error as SD. *p*-values were determined by unpaired *t*-test. *: *p* ≤ 0.05, ***: *p* ≤ 0.001. (**B**) MyC22-1, -2, or -3-bearing mice were treated with HF Moxetumomab injections as in (**A**). Untreated mice were euthanized at treatment start and treated mice one day after the last injection. Tumor infiltration was analyzed in BM, Spl, and lymph nodes (LN) by flow cytometry after staining with anti-human CD22 and anti-mouse CD19. Bars show means of n = 2–3 mice; each symbol represents an individual mouse. Shown are representative results of at least two independent experiments. Error as SD. *p*-values were determined by unpaired *t*-test. **: *p* ≤ 0.01, ***: *p* ≤ 0.001. (**C**) MyC22-2 was injected on day one and mice were treated with two doses of 200 µg anti-PD-L1 and with HF Moxetumomab alone or in combination (Combi) at the indicated time points. Shown are Kaplan-Meier survival curves with group sizes of n = 3–8 mice. *p*-values were determined by log rank test. *: *p* ≤ 0.05.

Next, we tested functionality of the novel chimeric h/mCD22 as target for human CD22-directed therapies using the immunotoxin Moxetumomab pasudotox. Moxetumomab is more active against human xenografts in mice when given repeatedly or continuously, which results in maintained drug serum levels over time in contrast to the clinical standard of bolus doses [32,33,34]. To be able to compare our new model with these earlier findings, we injected Moxetumomab at the same total dose of 1.2 mg/kg either as bolus or at high frequency (HF) in MyC22-2-bearing mice (Figure 6A). The bolus group received three doses of 0.4 mg/kg i.v. and the HF group received 0.1 mg/kg Moxetumomab i.p. every three hours, four times per day on three consecutive days (Figure 6A). We chose HF over pump implantation to avoid immune stimulation of the immunocompetent mice by abdominal surgery. In MyC22-2, bolus treatment decreased tumor burden in BM and spleen by 2.5-fold (Figure 6A). In contrast, Moxetumomab given at HF reduced lymphoma infiltration rate in the BM on average by 93-fold and in spleen on average by 20-fold which was significantly higher compared to bolus injection (*p* = 0.0405 for BM; *p* = 0.0003 for spleen; Figure 6A). Because lower efficacy of Moxetumomab was shown after bolus treatment, we next tested efficacy of HF treatment in all three MyC22 models. Moxetumomab consistently reduced tumor infiltration rate of all three clones in BM to less than 1% (*p* = 0.0075 for MyC22-1, *p* = 0.0006 for MyC22-2, *p* = 0.0005 for MyC22-3) and in spleen to less than 2% (*p* = 0.0002 for MyC22-1, *p* = 0.0007 for MyC22-2, *p* = 0.0009 for MyC22-3; Figure 6B). Different from the similarly high efficacy in BM and spleen of all three models, efficacy of Moxetumomab in LNs varied. HF treatment reduced LN infiltration of MyC22-1 from 3.8% to less than 0.1% (*p* = 0.0003), stabilized LN infiltration of MyC22-3 at 25.6%, and LN infiltration of MyC22-2 progressed from 5.8% to 25.1% within four days from treatment start (Figure 6B). Because all three lymphoma cell clones were similarly sensitive to Moxetumomab in BM and spleen, local TME was likely responsible for the distinct response patterns observed in LN.

### 2.5. Anti-PD-L1 Blockade Enhances Survival of Moxetumomab Treated Mice

Finally, we determined efficacy of CD22-targeted Moxetumomab in combination with immune modulation using anti-PD-L1 against MyC22-2 (Figure 6C). Anti-PD-L1 was given twice per week starting one week prior to Moxetumomab HF treatment (Figure 6C). Untreated MyC22-2-bearing mice survived on average 22.5 days. Anti-PD-L1 treatment alone did not result in a significant survival benefit with a median survival of 23 days (Figure 6C and Figure S4). Three day treatment of Moxetumomab alone prolonged survival on average by 2.5 days to 25 days compared to untreated controls (*p* = 0.0389). The combination of anti-PD-L1 and Moxetumomab significantly prolonged survival to a median of 28.5 days compared to Moxetumomab alone (*p* = 0.0380).

## 3. Discussion

While subcutaneous lymphoma was infiltrated by only few immune cells, systemically growing lymphoma presented with lymphoid organ-specific immune infiltration, thus resembling human disease more closely. As expected from an immune competent model of an aggressive B cell lymphoma, myeloid cells were of immunosuppressive phenotype characteristic for MDSCs which, in addition, supported lymphoma growth in BM. While anti-PD-L1 therapy alone was inefficient, CD22-targeted therapy with the immunotoxin Moxetumomab was highly active in BM and spleen and was further enhanced by anti-PD-L1 immune checkpoint inhibition.

The major goal of our study was to establish an immune-competent, primary murine lymphoma model for treatment evaluation of drugs against human CD22 in an immunocompetent TME that closely reflected human disease. Subcutaneous tumor models are a comparatively fast and simple model system to assess drug efficacy. Different from systemically growing lymphoma, s.c. lymphoma in our hands showed little immune infiltration and thus does not represent the commonly found TME of aggressive human lymphoma. The three distinct lymphoma clones displayed different growth kinetics in lymphoid organs and showed clone-specific immune infiltration even though all three clones share the same *myc* translocation as oncogenic driver. It is likely that distinct cooperative mutations of *myc*-driven lymphoma are responsible for some of the differences in phenotype [35,36]. Although distinct from each other, immune infiltration at organ sites remained similar to the immune cell composition of healthy mice. In line, T cells dominated the TME of LN while myeloid cells dominated the TME of BM, thus mirroring the most common immune cell type under physiologic conditions in the respective organ. Resemblance of physiologic, tissue-specific TME may be of relevance to produce results with predictive value for clinical translation, highlighting possible usefulness of our model system [4,37,38]. Within lymphoma TME, myeloid cells play a key role [9]. High numbers of MDSCs predict poor prognosis, suppress T cells in the local TME, and support lymphoma growth [13,14,15,16,39]. As expected from human MDSCs in B cell lymphoma, we found that Gr-1^+^ myeloid cells were of immunosuppressive phenotype and contributed to enhanced systemic lymphoma growth in immunocompetent mice as verified by depletion experiments. Although Gr-1 can be expressed on a subset of pre/pro B cells accounting for approximately 0.03% of murine BM [40], we did not find expression of Gr-1 on our lymphoma cells. Hence, observed effects on reduced lymphoma growth is likely due to targeting Gr-1^+^ myeloid cells, which are known for supporting B cell lymphoma rather than elimination of a subset of early B cells. Taken together, our results on MDSCs further support resemblance of the human disease suggesting a possible predictive strength of this unique mouse model for clinical translation.

Even though PD-L1 was highly expressed in TME of the systemic lymphoma model, anti-PD-L1 treatment was not effective. This is in line with only a small number of patients with aggressive B cell lymphoma benefitting from immune checkpoint blockade [31,41,42]. In contrast to our findings, immune checkpoint inhibitors are more active as a single agent as well as in combination with immunotoxins in preclinical models of solid tumor [43,44]. In these models of subcutaneously growing carcinoma, complete remission is potently induced after intratumoral injections of immunotoxins combined with checkpoint molecules and is accompanied by increased CD8^+^ T cell infiltration of the s.c. tumors. The depletion of CD8^+^ T cells reduces response rates dramatically indicating that adaptive immune responses—presumably based on recognition of tumor antigens—are needed for durable complete remission following checkpoint therapy [44]. In contrast, we did not detect substantial changes in T cell phenotype, T cell activation, change of CD4/CD8 T cell ratio, or induction of durable remission. This difference in responsiveness in our lymphoma model compared with studies using immunotoxin and checkpoint molecules against mesothelioma may be explained by the low mutational burden of lymphoma that therefore likely lack T cell epitopes needed for an adaptive immune response and thus the induction of durable remission [45,46,47]. Furthermore, B cell lymphoma are strong inducers of MDSCs which in turn suppress T cell function [48]. Hence, immune activation by anti-PD-L1 may not be sufficient to efficiently activate T cells against lymphoma in the immunosuppressive TME.

Moxetumomab traverses various intracellular compartments before it ADP-ribosylates eEF2. As such, we chose Moxetumomab to test functionality of the chimeric h/mCD22 [25]. We found Moxetumomab to be highly active against the three distinct, *myc*-driven lymphoma clones expressing h/mCD22, suggesting appropriate intracellular trafficking of h/mCD22. In human xenograft models of B ALL, of Burkitt’s lymphoma, of DLBCL, and of mantle cell lymphoma, responses to CD22-targeted immunotoxins were greatly enhanced by repeated or continuous administration via surgically implanted osmotic pumps [32,33]. In line with this efficacy of Moxetumomab in immunocompromised xenografts of human lymphoma, HF injections were more active than bolus doses in our h/mCD22^+^, primary murine lymphoma, thus indicating functional similarity of human CD22^+^ lymphoma and the newly established h/mCD22^+^ murine lymphoma. After observing similarly strong responses in BM and spleen, we were surprised that responses in LN strongly varied between the three models. This variability is probably not explained by drug accessibility to LN per se, because MyC22-1 was efficiently eliminated from LN. Since Moxetumomab was very active against lymphoma of all three clones when growing in BM and spleen, our findings may indicate that therapeutic efficacy in LN is influenced by organ-specific TME, further underlining the importance of drug tests in a systemic rather than a subcutaneous model [4,38,49,50,51,52,53].

In summary, we have generated the first lymphoma model for testing drugs against human CD22 in an immunocompetent mouse. The model resembles human disease closely and may be of great value for future testing of antibody-based therapeutics towards human CD22 including ADCs, immunotoxins, or CAR-T cells, alone or in combination with immune-modulatory drugs. Similar advantages of a systemic disease model may be applicable for other human molecules, including CD19 and CD20.

## 4. Materials and Methods

### 4.1. Mice

Animal studies were approved by the Institutional Animal Care and Use Committee. Animals were handled according to institutional guidelines.

C57BL/6 mice or NSG mice (NOD.Cg-*Prkdc*^*scid*^ *Il2rg*^*tm1Wjl*^/SzJ) were used. Homozygous h/mCD22 mice were crossbred with heterozygous λ-myc mice. Offspring of F_1_ generation were backcrossed with homozygous h/mCD22 mice (F_0_) to achieve mice homozygous for h/mCD22 and heterozygous for λ-myc in F_2_ generation [25,29]. These mice developed systemic h/mCD22^+^ murine B cell lymphoma at six weeks of age.

### 4.2. Systemic and Subcutaneous Tumor Growth

H/mCD22^+^ lymphoma were isolated from LNs of h/mCD22^+/+^ x λ-myc^+/−^ mice. Single cell suspensions were viably frozen and expanded by serial transplantation in syngeneic h/mCD22 mice. Prior to injection, thawed cells were washed twice and resuspended in 200 µL PBS (Gibco™, Thermo Fisher Scientific, Waltham, MA, USA). For systemic tumor growth, one million lymphoma cells were injected in the tail vein and for subcutaneous growth, ten million cells were injected s.c. in the upper flank of syngeneic mice. Tumor infiltration of systemically injected mice was measured postmortem by flow cytometry. S.c. tumor growth was measured by caliper and tumor volume was calculated as length × width^2^ × 0.4.

### 4.3. Histology

Lymphoma-infiltrated organs were fixed in 10% neutral-buffered formalin for 24–48 h, embedded in paraffin and stained by hematoxylin and eosin (H&E). Slides were scanned using the PANNORAMIC 250 slide scanner (3DHistech, Budapest, Hungary) and analyzed with CaseViewer 2.4 (3DHistech).

### 4.4. Flow Cytometric Analysis

Single cell suspensions of LN and spleen were prepared by meshing organs through a 70 µm cell strainer. BM was extracted by flushing femurs prior to meshing. Cells were washed with PBS (Gibco™, Thermo Fisher Scientific) and stained with Zombie Aqua (BioLegend, Amsterdam, The Netherlands), Fc blocked (anti-murine CD16/32, BioLegend), and stained with fluorescent-labeled antibodies against CD22 (clone HIB22), CD19 (clone 6D5), CD11b (clone M1/70), Ly6C (clone HK1.4), Ly6G (clone 1A8), CD3 (clone 17A2), CD4 (clone GK1.5), CD8α (clone 53-6.7), PD-1 (clone RMP1-30), all from BioLegend, and PD-L1 (clone MIH5) from Invitrogen^®^ (Thermo Fisher Scientific). Fluorescent-labeled rat IgG2b, κ isotype control antibody (clone RTK4530), and rat IgG2a, κ isotype control antibody (clone RTK2758) were from BioLegend. For intracellular IFNγ staining, cells were treated with Brefeldin A (BioLegend) for 4 h at 37 °C and stained with Zombie Aqua and antibodies. Then, cells were fixed (Fixation Buffer, BioLegend), permeabilized (Permeabilization Wash Buffer, BioLegend), stained with anti-IFNγ (clone XMG1.2, BioLegend), washed, and measured by flow cytometry using FACSCantoII (BD, Franklin Lakes, NJ, USA). Data was analyzed by FlowJo (Tree Star, Ashland, OR, USA). All flow cytometry data was gated on single cell events (FSC-H/FSC-W, SSC-A/SSC-H), of which debris and red blood cells were excluded by FSC-A/SSC-A, and dead cells were stained and excluded as Zombie Aqua positive. Living single cells were analyzed for indicated markers. Monocytic MDSCs were gated as CD11b^+^Ly6C^high^Ly6G^−^ and granulocytic MDSCs as CD11b^+^Ly6C^low^Ly6G^+^.

### 4.5. T Cell Suppression Assay

For in vitro co-culture experiments, spleens of MyC22-2-bearing mice and of healthy mice were digested with Liberase DL (Roche, Mannheim, Germany) and DNase I (Sigma-Aldrich^®^, Merck, Darmstadt, Germany) for 30 min at 37 °C. After stopping digestion with MACS buffer (HBSS (Carl Roth, Karlsruhe, Germany)/5% heat inactivated FCS (Sigma-Aldrich^®^, Merck)/5 mM EDTA (Millipore^®^, Merck), cell suspensions were meshed through a 70 µm cell strainer and treated with 1x RBC lysis buffer (eBioscience^®^, Thermo Fisher Scientific) for 3 min on ice. Myeloid cells were magnetically sorted from tumor-bearing spleen using CD11b^+^ microbeads (Miltenyi Biotec, Bergisch Gladbach, Germany). Untouched CD4^+^ T cells were isolated from healthy spleens by CD4^+^ T cell isolation kit (Miltenyi Biotec). Isolated T cells were stained with CFSE (BioLegend) and stimulated with anti-mouse CD3ε and CD28 MACSiBeads™ (Miltenyi Biotec) in a bead-to-cell ratio of 2:1. Isolated CD11b^+^ cells were added in ratios of 1:64 to 1:4. Cells were grown in RPMI (Gibco™, Thermo Fisher Scientific) supplemented with 10% heat-inactivated FCS (Sigma-Aldrich^®^, Merck), 1% L-Glutamine, 100 U penicillin, 100 mg streptomycin (Gibco™, Thermo Fisher Scientific), 0.01 mM β-Mercaptoethanol (Sigma-Aldrich^®^, Merck). After 72 h of co-culture, T cell proliferation was analyzed by measuring CFSE^+^ cells by flow cytometry.

### 4.6. Immune Cell Depletion

For immune cell depletion, 200 µg of In Vivo MAb anti-mouse Gr-1 (clone RB6-8C5, BioXCell, Lebanon, NH, USA), anti-mouse CD4 (clone GK1.5, BioXCell), and anti-mouse CD8α (clone 2.43, BioXCell) antibodies or rat IgG2b isotype control (clone LTF-2, BioXCell) were injected i.p. in PBS (Gibco™, Thermo Fisher Scientific) on days 0, 3, 7, 10, 14. MyC22-2 cells were inoculated i.v. on day one as described earlier. On day 15, mice were humanely euthanized, and BM was analyzed by flow cytometry.

### 4.7. Treatment Studies

Anti-mouse PD-L1 (MedImmune, Gaithersburg, MD, USA) was diluted in PBS (Gibco™, Thermo Fisher Scientific) and administered i.p. at a dose of 200 µg twice per week for one week. Moxetumomab (Lumoxiti^®^, MedImmune) was given either as bolus or as HF treatment. For bolus treatment, 0.4 mg/kg Moxetumomab, dissolved in PBS (Gibco™, Thermo Fisher Scientific), was injected i.v. as three single bolus doses. For HF, the 0.4 mg/kg Moxetumomab was split into four equal doses of 0.1 mg/kg and injected i.p. every three hours for three consecutive days. To analyze tumor response and immune infiltration, mice were euthanized at indicated time points. For survival studies, mice were observed until disease progression which was determined by a predefined scoring system according to the approved animal protocol. Mice reaching a score of 20 points fulfilled termination criteria and were humanely sacrificed. The score included weight loss (10–20% = 10 points, >20% = 20 points), changes in animal behavior or appearance (between 5 and 20 points), and beginning/mild hind limb paralysis (20 points).

### 4.8. Statistical Analysis

Data were analyzed in GraphPad Prism v8.3.0 (GraphPad Software, San Diego, CA, USA). Statistical significance of two groups was determined by *t*-test and multiple comparisons were performed by one-way ANOVA (Dunnett’s test) as indicated. For survival analyses, log rank tests were used.

## Data Availability

Not applicable.

## Supplementary Materials

Supplementary materials are available online at https://www.mdpi.com/article/10.3390/ijms221910433/s1.
