# Supplementary material for: Human CD22-Transgenic, Primary Murine Lymphoma Challenges Immunotherapies in Organ-Specific Tumor Microenvironments
Source: Int J Mol Sci. 2021 Sep 28;22(19):10433. doi: 10.3390/ijms221910433 (PMC8508822; doi:10.3390/ijms221910433)
Supplement: Supplementary file 1 [file ijms-22-10433-s001.zip › ijms-1381242-supplementary.pdf]

Supplementary Files

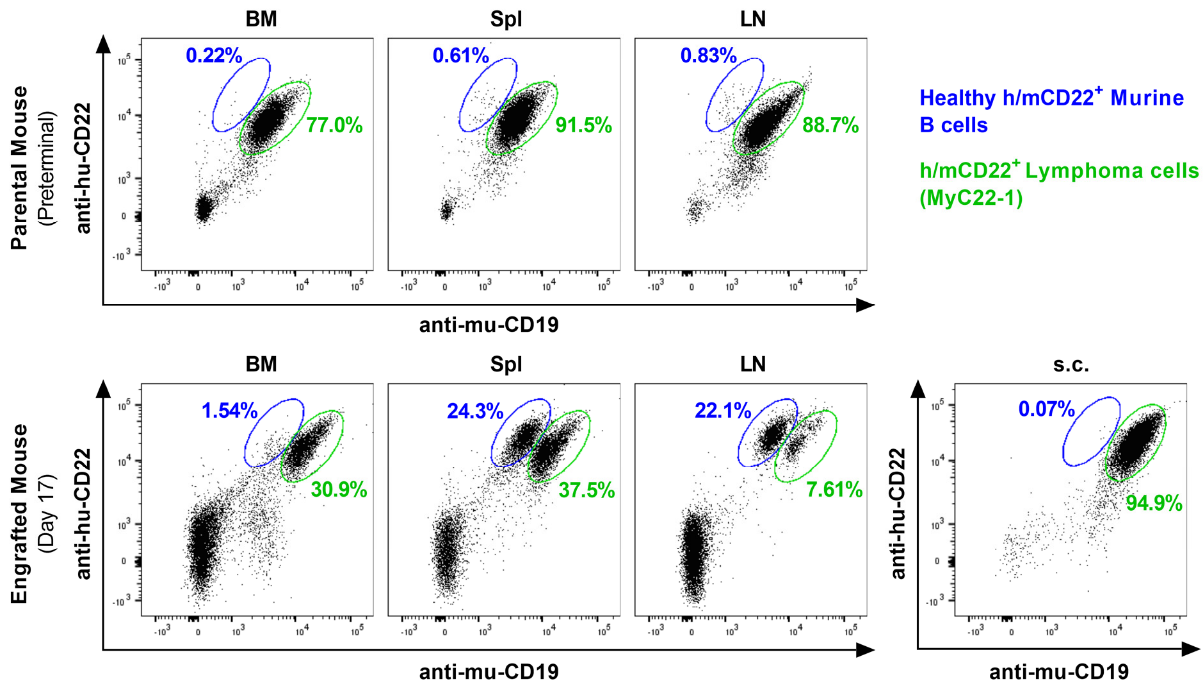

Supplementary Figure S1. Healthy and malignant B cells of immunocompetent, MyC22–1–bearing mice. λ–myc$^{+/-}$ h/mCD22$^{+/+}$ mice, which spontaneously developed lymphoma, were euthanized when reaching the defined criteria according to institutional guidelines and the approved animal protocol. Cells from bone marrow (BM), spleen (Spl), and lymph nodes (LN) were isolated, viably frozen and reinjected in syngeneic h/mCD22$^{+/+}$ mice either intravenously or subcutaneousely (s.c.). On day 17 after re–injection, otherwise healthy mice were sacrificed and BM, Spl, and LN from intravenously injected mice and s.c. tumors from s.c. injected mice were extracted. Murine CD19^{+} (mu–CD19) and human CD22^{+} (hu–CD22) B cells were analyzed by flow cytometry. Shown are representative dot blots for MyC22–1. Gates indicate healthy h/mCD22^{+} murine B cells (blue circle) and MyC22–1 lymphoma cells (green circle).

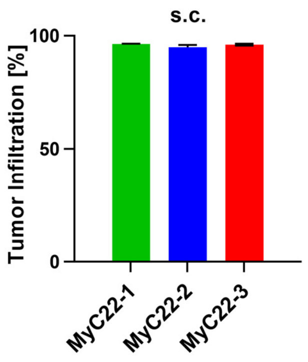

Supplementary Figure S2. Tumor infiltration of subcutaneously growing MyC22. Subcutaneous (s.c.) MyC22–1, –2, –3 tumors were extracted at a size of ~400 mm^3. Tumor infiltration was analyzed by flow cytometry after staining with anti–human CD22 and anti–mouse CD19. Bars show means of n=3 mice. Error as SD.

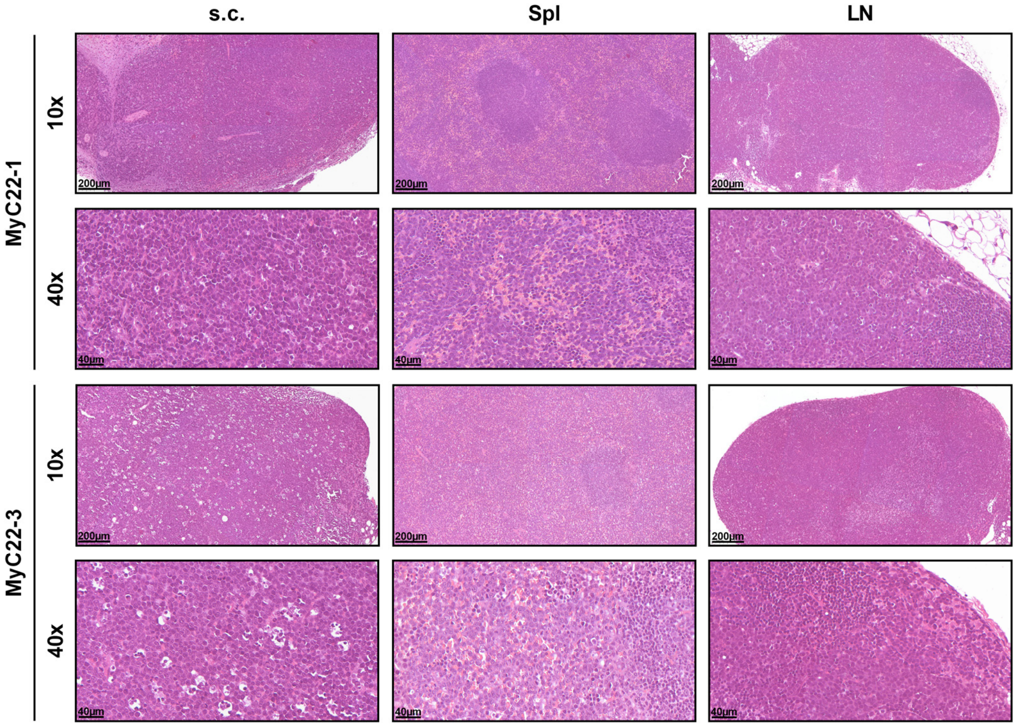

Supplementary Figure S3. Histological characterization of lymphoma–infiltrated organs. Subcutaneous (s.c.) tumors of MyC22–1 and of MyC22–3 were extracted at a size of ~400 mm³. Systemically infiltrated spleen (Spl) and lymph nodes (LN) were extracted on day 17 of MyC22–1 and on day 20 of MyC22–3. Shown are representative sections of indicated organs that were fixed and stained with hematoxylin and eosin (H&E). Shown are magnifications of 10–fold and 40–fold.

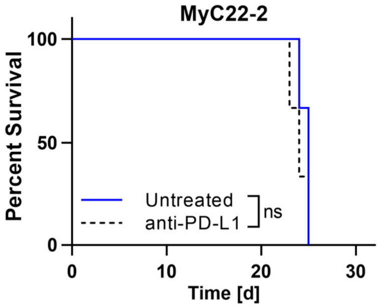

Supplementary Figure S4. Immune checkpoint blockade with anti–PD–L1 does not prolong survival of MyC22–2–bearing mice. MyC22–2 was injected on day one and mice were treated with two doses of 200 µg anti–PD–L1 on days 15 and 17. Shown are Kaplan Meier survival curves with a group size of n=3 mice. P–values were determined by log–rank test. Not significant (ns): $p > 0.05$.
